# *MLO* Differentially Regulates Barley Root Colonization by Beneficial Endophytic and Mycorrhizal Fungi

**DOI:** 10.3389/fpls.2019.01678

**Published:** 2020-01-16

**Authors:** Magdalena Hilbert, Mara Novero, Hanna Rovenich, Stéphane Mari, Carolin Grimm, Paola Bonfante, Alga Zuccaro

**Affiliations:** ^1^ Department of Organismic Interactions, Max Planck Institute of Terrestrial Microbiology, Marburg, Germany; ^2^ Department of Life Sciences and Systems Biology, University of Turin, Turin, Italy; ^3^ Botanical Institute, Cluster of Excellence on Plant Sciences (CEPLAS), University of Cologne, Cologne, Germany; ^4^ BPMP, Univ Montpellier, CNRS, INRAE, Montpellier SupAgro, Montpellier, France

**Keywords:** biotrophy, cell death, fungal-root interactions, mutualism, cell wall appositions, Perls/DAB, VPE activity, susceptibility gene

## Abstract

Loss-of-function alleles of *MLO* (*Mildew Resistance Locus O*) confer broad-spectrum resistance to foliar infections by powdery mildew pathogens. Like pathogens, microbes that establish mutually beneficial relationships with their plant hosts, trigger the induction of some defense responses. Initially, barley colonization by the root endophyte *Serendipita indica* (syn. *Piriformospora indica*) is associated with enhanced defense gene expression and the formation of papillae at sites of hyphal penetration attempts. This phenotype is reminiscent of *mlo*-conditioned immunity in barley leaf tissue and raises the question whether *MLO* plays a regulatory role in the establishment of beneficial interactions. Here we show that *S. indica* colonization was significantly reduced in plants carrying *mlo* mutations compared to wild type controls. The reduction in fungal biomass was associated with the enhanced formation of papillae. Moreover, epidermal cells of *S. indica*-treated *mlo* plants displayed an early accumulation of iron in the epidermal layer suggesting increased basal defense activation in the barley mutant background. Correspondingly, the induction of host cell death during later colonization stages was impaired in *mlo* colonized plants, highlighting the importance of the early biotrophic growth phase for *S. indica* root colonization. In contrast, the arbuscular mycorrhizal fungus *Funneliformis mosseae* displayed a similar colonization morphology on mutant and wild type plants. However, the frequency of mycorrhization and number of arbuscules was higher in *mlo-*5 mutants. These findings suggest that *MLO* differentially regulates root colonization by endophytic and AM fungi.

## Introduction

Plants establish diverse beneficial interactions with fungi from different taxa. Root endophytes belonging to the order Sebacinales establish long-lasting beneficial relationships with a broad range of plant species (Weiss et al., 2016). Root colonization by members of this order results in enhanced growth (Varma et al., 1999; Waller et al., 2005; Serfling et al., 2007; Ghimire et al., 2009; Franken, 2012), improved tolerance to abiotic stress (Waller et al., 2005; Baltruschat et al., 2008; Sherameti et al., 2008; Ghimire and Craven, 2011), as well as increased resistance to pathogens (Stein et al., 2008; Waller et al., 2008; Lahrmann et al., 2015; Sarkar et al., 2019). In some cases nutrient status has been reported to play a role in the interaction of the model sebacinoid fungus *Serendipita indica* with some plant species (Shahollari et al., 2005; Sherameti et al., 2005; Nautiyal et al., 2010; Yadav et al., 2010; Kumar et al., 2012). However, *S. indica* colonization of *Nicotiana attenuata* and barley had no effect on host phosphorus (P) and nitrogen (N) content (Barazani et al., 2005; Achatz et al., 2010), suggesting that nutrient exchange is not central to the beneficial effects conferred by sebacinoid fungi. *S. indica* colonizes the rhizodermis and outer root cortex (Deshmukh et al., 2006; Jacobs et al., 2011; Weiss et al., 2016). Following an initial biotrophic growth phase, during which the fungal hyphae remain surrounded by a plant-derived membrane, *S. indica* transitions to cell death-associated colonization that does not result in host disease (Deshmukh et al., 2006; Jacobs et al., 2011; Zuccaro et al., 2011; Lahrmann and Zuccaro, 2012; Qiang et al., 2012; Lahrmann et al., 2013).

Like sebacinoid fungi, arbuscular mycorrhizae (AM) establish beneficial interactions with many plant species (Bonfante and Genre, 2010). AM are obligate biotrophs that rely on their plant hosts as carbon sources in exchange for soil nutrients including P and N (Parniske, 2008). Additionally, AM colonization results in increased plant biomass and confers enhanced resistance to stress and pathogen infection (Khaosaad et al., 2007; Liu J et al., 2007; Pozo and Azcon-Aguilar, 2007). Following the mutual recognition between plant and microbe, AM form specialized hyphae, called hyphopodia, that adhere to the root epidermal surface where penetration hyphae emerge. In the inner root cortex, intracellular hyphae then establish so-called arbuscules, which represent the active interface for nutrient exchange (Genre et al., 2008).

The successful establishment of AM symbioses is genetically controlled by the ancestral common symbiosis pathway (CSP). In legumes, this pathway is required for the establishment of AM as well as root nodule symbiosis with rhizobacteria (Kistner et al., 2005; Gutjahr, 2014; Svistoonoff et al., 2014). In contrast, *S. indica* colonization and development is independent of *Lotus japonicus* and *Arabidopsis thaliana* CSP genes, suggesting that independent host pathways control AM symbiosis and endophytism (Banhara et al., 2015). However, in both cases transient and weak activation of defense responses have been reported during the early phases of colonization that are effectively suppressed by the fungi as the symbioses progress (Harrison, 2005; Schäfer et al., 2009; Camehl et al., 2011; Jacobs et al., 2011). These early defense responses include the formation of papillae at sites of hyphal penetration attempts of *S. indica* on barley (Lahrmann and Zuccaro, 2012). Papillae are dome-shaped cell wall appositions that play a vital role in resistance to plant pathogens (Hückelhoven, 2005; Hückelhoven, 2007; Albersheim et al., 2011; Hückelhoven, 2014). They generally consist of layers of callose, cellulose, arabinoxylan and phenolyic compounds (Chowdhury et al., 2014; Hückelhoven, 2014). Additionally, papillae contain reactive oxygen species (hydrogen peroxide H_2_O_2_) and in barley their formation appears to be dependent on iron (Fe^3+^) accumulation in the apoplast (Thordal-Christensen et al., 1997; Hückelhoven et al., 1999; Liu G et al., 2007). Depending on their size, composition and the degree of cross-linking of their constituent parts, papillae can be more or less efficient in halting penetration (Aist and Israel, 1977; Israel et al., 1980; von Röpenack et al., 1998; Asaad et al., 2004; Chowdhury et al., 2014; Hückelhoven, 2014).

In barley, natural as well as chemically induced mutant lines carrying recessive *mlo* (MILDEW RESISTANCE LOCUS O) alleles display broad-spectrum resistance to the obligate biotrophic pathogen *Blumeria graminis* f. sp. *hordei* (*Bgh*), causal agent of the foliar powdery mildew disease (Büschges et al., 1997), and have been successfully employed in agriculture since the late 1970s (Jørgensen, 1992; Lyngkjær et al., 2000; Kusch and Panstruga, 2017). Especially barley spring varieties that are now largely grown in central Europe are resistant to *Bgh* following the introgression of *mlo* alleles (Jørgensen, 1992; McGrann et al., 2014; Kusch and Panstruga, 2017). Compared to wild type susceptible cultivars, these lines display faster formation of larger papillae upon pathogen attack (Jørgensen, 1992; Lyngkjær et al., 2000; Chowdhury et al., 2014). Similar mutations in orthologous genes of wheat, tomato, pea, *A. thaliana*, and many other plant species have since confirmed the importance of *mlo* for resistance to various species of powdery mildew (Elliott et al., 2002; Consonni et al., 2006; Bai et al., 2008; Humphry et al., 2011; Wang et al., 2014; Kusch and Panstruga, 2017). In contrast, some hemibiotrophic and necrotrophic pathogens show enhanced infection on *mlo* mutant plants possibly profiting from the spontaneous induction of leaf cell death (Jarosch et al., 1999; Kumar et al., 2001; McGrann et al., 2014). The contribution of *MLO* to host resistance against root-colonizing microbes is less well understood. Recent evidence suggests that the *mlo* genetic background does not affect barley root infection by the oomycete pathogen *Phytophthora palmivora* (Le Fevre et al., 2016). Similarly, *MLO* does not seem to play a role in the establishment of the beneficial relationships between pea and the rhizobacterium *Rhizobium leguminosarum* bv. *viciae* or the AM fungus *Rhizophagus irregularis* (syn. *Glomus intraradices*) (Humphry et al., 2011). However, transcriptional analyses showed an upregulation of the *Lotus japonicus MLO1-like* (chr1.CM0150.1) gene in cortical cells containing arbuscules of *Gigaspora margarita* (Guether et al., 2009) suggesting that *MLO* may play a regulatory role in AM colonization.

In this study, we used the endophyte *S. indica* and the AM fungus *F. mosseae*, both of which have intracellular lifestyles, to investigate the role of *MLO* in barley root symbioses. Comparative colonization analyses showed differential regulation by *MLO* during endophytism and mycorrhization. The decreased colonization by *S. indica* coincided with enhanced defense responses and papillae formation, highlighting the importance of the biotrophic growth phase for the establishment of the long-term beneficial relationship between *S. indica* and its plant hosts.

## Materials and Methods

### Plant Material and *S. indica* Growth Conditions

Seeds of *mlo-*3, *-*4, and *-*5 mutant lines backcrossed to barley cv. Ingrid (Peterhänsel et al., 1997; Jarosch et al., 2003) were kindly provided by Ralph Panstruga. Wild type (WT) barley (*Hordeum vulgare* L. cv. Ingrid) and mutant seeds were surface-sterilized by washing in 70% ethanol for 1–5 min, rinsing in sterile distilled water and soaking in 4%–12% sodium hypochlorite for 1–1.5 h. Seeds were then thoroughly washed in sterile distilled water, placed onto sterile wet filter paper and kept in the dark at room temperature for 3–5 days to allow germination. *S. indica* Sav. Verma, Aj. Varma, Rexer, G. Kost & P. Franken (DSM11827, Deutsche Sammlung von Mikroorganismen und Zellkulturen, Braunschweig, Germany) was grown in liquid complex medium (CM) (Pham et al., 2004) at 130 rpm or on CM medium supplemented with 1.5% agar at 28°C.

### Barley Inoculation With *S. indica*

Three-day-old seedlings were placed into sterile jars containing 1/10 plant nutrition medium (PNM) (Basiewicz et al., 2012). For inoculation, *S. indica* chlamydospores were collected from CM agar plates in 0.1% Tween20-water, filtered through Miracloth and pelleted by centrifugation at 3,500 *g* for 5 min. Spores were washed two more times in 0.1% Tween20-water and then re-suspended to a final concentration of 5x10^5^ spores/ml. Mock treatment consisted of 0.1% Tween20-water only. Three milliliters of spore suspension was added onto roots of barley seedlings. Jars were transferred to a growth chamber with a 16 h/8 h day/night (light intensity of 108 µmol/m^2^/s) cycle at 22°C/18°C and 60% humidity. All experiments were prepared with three to four biological replicates consisting of pooled material from 4 plants/jar, and two to three independent replicate experiments.

### Quantification of *S. indica* Colonization

Mock-treated and *S. indica*-colonized roots were harvested at 3, 5, 7, and 10 days post inoculation. Roots were washed in water and sections of the first 3 cm below the seed were cut and frozen in liquid nitrogen. Genomic DNA from 200 mg of freshly ground material was isolated according to (Doyle and Doyle, 1987). To remove contaminating RNA, samples were treated with 1 µL 10 mg/mL RNaseA (Thermo Fisher Scientific, Schwerte, Germany) and incubated at 37°C for 20 min. Quantitative PCR was performed with 10 ng gDNA template and primers targeting the *S. indica TEF* (*SiTEF*) the barley ubiquitin (*HvUBI*) genes (see **Supplementary Table 1**) in 10 µl SYBR green Supermix (BioRad, Munich, Germany) using the following amplification protocol: initial denaturation for 16 min at 95°C, followed by 40 cycles of 15 s at 95°C, 20 s at 59°C, and 30s at 72°C, and a melt curve analysis. The relative amount of fungal *vs.* plant gDNA was calculated according to the 2^-Δ^ *^Ct^* method (Schmittgen and Livak, 2008).

### Vacuolar Processing Enzyme (VPE) Activity Assay

Mock-treated and *S. indica*-colonized roots of WT and mutant plants were harvested at 10 dpi. Vacuolar processing enzyme (VPE) activity was measured as described previously (Lahrmann et al., 2013). Briefly, roots were washed in water and sections of the first 4 cm below the seed were cut and frozen in liquid nitrogen. Extracts were prepared from 100 mg freshly ground root material ground in liquid nitrogen with 1 ml extraction buffer [10 mM sodium acetate pH 5.5, 100 mM NaCl, 1 mM EDTA, 2mM dithiothreitol (DTT) and 1mM phenylmethylsulfonyl fluoride (PMSF)]. Plant debris was pelleted by centrifugation at max speed and 4°C for 10 min. To measure VPE activity, 100 µM of the fluorescent VPE-specific substrate Ac-ESEN-MCA (Peptide Institute Inc., Osaka, Japan) was added to 100 µl of root extract supernatants aliquoted into a 96-well plate. Fluorescence intensities were measured in a TECAN Infinite microplate reader (TECAN, Männerdorf, Switzerland) with 360 and 465 nm excitation and emission wavelengths, respectively, at 10 min intervals for 1 h. Buffer with and without substrate was used as control.

### Staining and Microscopy of *S. indica*-Inoculated Barley Root Sections

Confocal pictures were taken using a TCS-SP5 confocal microscope (Leica, Bensheim, Germany). Colonized root tissue of WT and mutant plants was collected at indicated time points, boiled for 2 min in 10% potassium hydroxide, washed three times in deionized water, and three additional times in 1x PBS (pH 7.4) for 30 min. Roots were stained by infiltrating colorants four times for 4 min at 260 mbar with 1 min atmospheric pressure breaks. To visualize fungal structures, roots were infiltrated with 10 μg/mL fluorescent Wheat Germ Agglutinin (WGA) AF488 (Invitrogen, Thermo Fisher Scientific, Schwerte, Germany) in 1x PBS. Papillae were visualized following infiltration of 10 μg/mL fluorescent concanavalin A (ConA) AF633 (Invitrogen, Thermo Fisher Scientific, Schwerte, Germany) in 1x PBS. For iron staining, samples were fixed, embedded in resin, sectioned and then stained with the Perls/DAB procedure, as described previously (Roschzttardtz et al., 2009). This method is a sensitive histological test for iron accumulation in plants (Roschzttardtz et al., 2013).

### Barley Inoculation With the Mycorrhizal Fungus *F. mosseae*

Following germination on wet filter paper, seedlings were kept 3 days in continuous light (light intensity of 80 µmol/m^2^/s). For each experiment, four seedlings of barley WT and *mlo-*5 were placed into separate pots filled with a 7:3 mix of sterile quartz sand/granular *F. mosseae* inoculum (v/v). The *F. mosseae* inoculum was composed of newly formed spores and Sorghum root pieces already colonized by *F. mosseae*. The inoculum was purchased from MycAgro Lab (Dijon, France) and contained a minimum of 10 active propagules/g. Plants were transferred to a growth chamber with a 14 h/10 h day/night cycle at 23°C/21°C. After 2 months of cultivation, one half of the root apparatus was sampled from each plant and stained with Cotton Blue (0.1% in lactic acid) to evaluate the intraradical colonization. The other half was treated with WGA conjugated with the fluorescent probe fluorescein isothiocyanate (FITC) to analyze fungal penetration in the root epidermal cells by confocal microscopy. The experiment was performed twice.

### Quantification of the AM Colonization

For each plant at least 1 m of root tissue was observed under an optical microscope to evaluate the degree of mycorrhizal colonization (Trouvelot et al., 1986). Four parameters were considered: F% (frequency of mycorrhization) reporting the percentage of segments showing internal colonization, M% (intensity of mycorrhization in the root cortex) indicating the average percentage of colonized root segments, a% (percentage of arbuscules in the infected areas) quantifying the average presence of arbuscules within the infected areas, A% (percentage of arbuscules in the entire root apparatus) quantifying the presence of arbuscules in the whole root system.

### Details of the AM Penetration

To analyze the fungal penetration through the root epidermis, fifty 0.5 cm root segments were fixed in 4% paraformaldehyde in 0.05 M phosphate buffer pH 7.2, for 4 h at room temperature. Fixed segments were embedded in 8% agarose (Agarose type II-A, Sigma-Aldrich, Taufkirchen, Germany) and cut into 50 µm sections with a vibratome. The sections were incubated for 5 min in 1:30 commercial bleach/phosphate buffer (v/v), carefully rinsed with buffer and incubated for 2 h at room temperature in 1 mg/ml WGA-FITC. Sections were then observed with a Leica TCS SP2 confocal microscope equipped with an Ar/HeNe laser with 543 nm excitation and 580–650 nm emission wavelengths.

### Statistical Analyses

Statistically significant effects of plant genotypes on VPE activity in colonized and mock-treated root tissue were determined with ANOVA using R (v3.3.2). Significant differences between treatments were determined with Tukey’s *post hoc* test from the Stats package (Miller, 1981; Yandell, 1997). Letters displaying similarities and differences were extracted using the multcompView package (v0.1-7) (Donoghue, 2004; Piepho, 2004). Statistically significant effects of plant genotypes on fungal colonization and activation of defense responses (papillae size and number) were determined using the Welch Two Sample *t* test from the Stats package in R. Asterisks indicate these differences.

## Results and Discussion

### *MLO* Loss-Of-Function Mutations Result in Reduced Barley Root Colonization by *S. indica*

**Figure 1:**
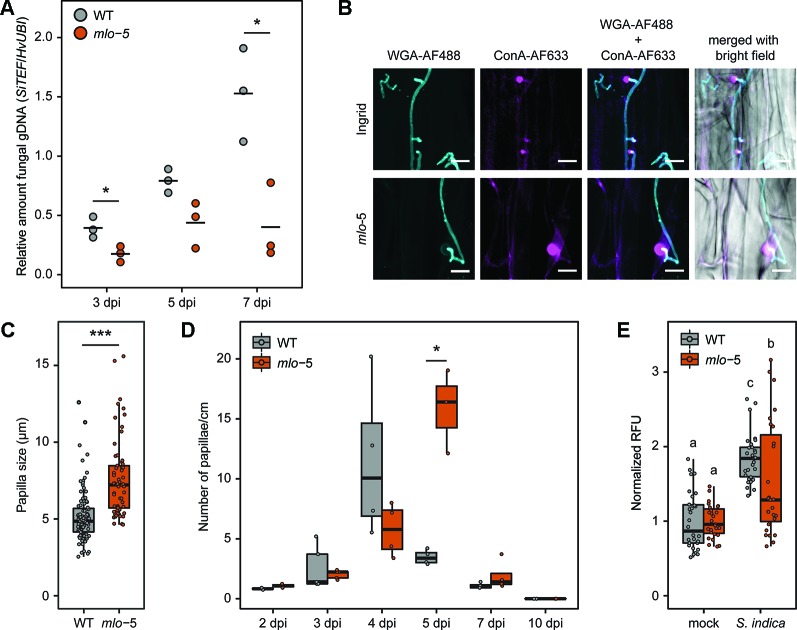
Reduced *S. indica* colonization of barley *mlo*-5 coincides with enhanced root defense. Three-day-old barley seedlings were inoculated with a *S. indica* chlamydospore suspension at a final concentration of 5x10^5^ spores/ml in Tween20-water or Tween20-water alone as mock treatment. **(A)** At 3, 5, and 7 days post inoculation (dpi) seedlings were removed from jars and gDNA was extracted from inoculated root sections as described in the *Materials and Methods*. Fungal colonization in each biological replicate was confirmed by quantitative PCR (*n* = 3). Statistically significant differences in the relative abundance of fungal gDNA during colonization of wild type (WT) and mutant *mlo-*5 plants were determined with Welch two sample *t* tests (*p < 0.5). **(B)** Roots of inoculated WT and *mlo*-5 plants were collected and stained with 10 μg/ml Wheat Germ Agglutinin (WGA-AF488, cyan) and 10 μg/ml concanavalin A (ConA-AF633, magenta) for visualization of fungal structures and papillae, respectively. Confocal microscopy shows extraradical growth of *S. indica* and hyphal penetration attempts of WT and *mlo-*5 root tissue. At these sites, the barley host responds with papillae formation. **(C)** Sizes of papillae formed in colonized WT (*n*=95) and *mlo-*5 (*n* = 61) roots were determined based on confocal microscopy pictures taken at 3, 4, 5 and 7 dpi. The statistically significant difference in papilla size between wild type (WT) and mutant *mlo-*5 plants throughout colonization was determined with the Welch two sample *t* test (***p < 0.001). **(D)** The number of papillae formed in colonized WT and *mlo-*5 roots was quantified based on confocal microscopy pictures taken at 2, 3, 4, 5, 7, and 10 dpi (*n* = 2-4). Statistically significant differences in papilla quantity between barley genotypes were determined with Welch two sample *t* tests (*p < 0.5). **(E)** Mock-treated and *S. indica*-colonized roots of WT and mutant plants were harvested at 10 dpi. Root cell death was quantified by measuring vacuolar processing enzyme (VPE) activity-dependent fluorescence (Qiang et al., 2012). Letters represent statistically significant differences in VPE activity according to two-way ANOVA (*F*(1,110) = 7.077, *p* < 0.01) and Tukey’s *post hoc* test.

Removal of host genes that are required by invading pathogens for plant colonization, termed susceptibility genes, has been shown to provide disease resistance (van Schie and Takken, 2014). Several natural and mutagen-induced changes in the barley *MLO* locus confer broad-spectrum resistance to the powdery mildew pathogen *Blumeria graminis* f. sp. *hordei* (Jørgensen, 1992; Büschges et al., 1997; Lyngkjær et al., 2000; Kusch and Panstruga, 2017). Similarly, TALEN- and CRISPR-Cas9-introduced targeted mutations in three *MLO* homeoalleles of bread wheat showed their requirement for resistance to wheat powdery mildew (Wang et al., 2014). However, the recessive loss-of-function *mlo* alleles in barley resulted in increased susceptibility to some other fungal pathogens (Jarosch et al., 1999; McGrann et al., 2014). To investigate the role of *MLO* in the establishment of the long-term beneficial relationship between barley and the root endophyte *Serendipita indica*, we used the *mlo-*5 allele backcrossed (BC) into barley cv. Ingrid (hereafter referred to as *mlo-*5), which carries a point mutation in the start codon and is a predicted null allele (Büschges et al., 1997). Mutant and WT plants were inoculated with *S. indica* spores suspended in Tween20-water or Tween20-water alone as mock treatment. To assess the effect of the *mlo-*5 mutation on *S. indica* colonization, we quantified the relative abundance of *S. indica* gDNA as a proxy for fungal biomass in roots of WT barley cv. Ingrid and *mlo-*5 plants by quantitative PCR at 3, 5, and 7 days post inoculation (dpi), representing early and late biotrophic interaction stages and the initial cell death-associated phase, respectively. We observed a significant reduction in fungal biomass in mutant compared to control plants from early through late colonization stages (**Figure 1A**). To confirm this phenotype, we also quantified *S. indica* biomass in roots of colonized *mlo-*3 and *mlo-*4 mutant lines, which display frame shift mutations in exon 11 and 4, respectively, and likewise are predicted null alleles. Similar to *mlo-*5, *mlo-*3 and *-*4 were less colonized by *S. indica* compared to WT control plants (**Supplementary Figure 1**), suggesting that *MLO* is required for barley root colonization by *S. indica*. This is in contrast to earlier findings where *mlo* triple mutants of *A. thaliana* displayed similar levels of *S. indica* colonization as wild type plants (Acevedo-Garcia et al., 2017). In *A. thaliana* there are 15 members of the MLO family. It could be that in *A. thaliana* roots other MLO members play a role in fungal accommodation than the three mutated genes. Alternatively, it has previously been shown that *S. indica* displays different colonization strategies on barley and *A. thaliana* host plants (Lahrmann et al., 2013). It is, therefore, conceivable that MLO-controlled defense does not play an important role in *S. indica* accommodation in *A. thaliana* roots.

### *S. indica*-Colonized *mlo*-5 Mutant Plants Display Enhanced Papilla Formation During Biotrophic Growth

Enhanced barley resistance to powdery mildew in *mlo* mutants has been associated with enhanced cell wall apposition, or papillae, formation (Skou et al., 1984; Bayles et al., 1990; Wolter et al., 1993). Due to the reduction in *S. indica* colonization in *mlo* plants, we compared papillae formation in inoculated *mlo-*5 mutant and WT roots. To visualize fungal structures and papillae, colonized root tissue was stained with the chitin-specific wheat germ agglutinin (WGA-AF488) and α-mannopyranosyl-/α-glucopyanosyl- specific concanavalin A (ConA-AF633) for confocal microscopy, respectively. While both barley genotypes responded with papillae formation to *S. indica* hyphal penetration attempts, these cell wall appositions were significantly bigger in *mlo-*5 plants compared to the controls (**Figures 1B**, **C**). The number of papillae increased over time both in WT and *mlo-*5 plants but was significantly higher at 5 dpi in mutant plants (**Figure 1D**). These phenotypes are reminiscent of *mlo*-mediated resistance to *Bgh* in leaves, where the fungus is arrested at the prehaustorial stage when papillae formation occurs in epidermal cells (Skou et al., 1984), highlighting an interesting parallel between *mlo*-dependent leaf and root responses to fungal colonization.

At early stages of WT barley root colonization by *S. indica*, transient papillae development has previously been observed at penetration sites and coincides with the activation of weak barley defense responses at the transcriptional level during the fungus’ biotrophic growth phase (Schäfer et al., 2009; Zuccaro et al., 2011; Lahrmann and Zuccaro, 2012). However, most of the papillae formed during the biotrophic colonization of the rhizodermis do not prevent *S. indica* hyphal penetration and disappear once the fungus reaches the root cortex (Zuccaro et al., 2011; Lahrmann and Zuccaro, 2012). The role of papillae in resistance against host cell penetration has been discussed for decades (Zeyen et al., 2002). Recent evidence suggests that so-called effective papillae [cell wall appositions that are formed in response to a local colonization attempt and that cannot be penetrated; (Hückelhoven, 2014)] contain higher quantities of callose, arabinoxylan and cellulose than ineffective papillae in barley (Chowdhury et al., 2014). Noncovalent bonds between arabinoxylan and cellulose have been proposed to maintain the barley cell wall, potentially forming a network of highly cross-linked polysaccharides (McNeil et al., 1975; Chowdhury et al., 2014). Phenolic compounds present in papillae could further enhance the degree of cross-linking giving rise to a tight structure resistant to mechanical penetration. The resistance to mechanical force, however, is unlikely to play a role in the interaction between *S. indica* and barley, since *S. indica* does not form appressorium-like structures. The genome of *S. indica*, like the genome of its orchid mycorrhizal relative *S. vermifera*, harbors a large number of genes encoding hydrolytic enzymes comparable to genomes of hemibiotrophic and necrotrophic pathogens, as well as several white rot saprotrophs (Lahrmann et al., 2013; Lahrmann et al., 2015). The up-regulation of genes encoding putative cell wall degrading enzymes during the pre-penetration stage (Zuccaro et al., 2011) suggests that *S. indica* uses hydrolytic enzymes for host cell penetration. The phenotypic alteration of papillae formed in *mlo-*5 plant roots suggests a change in composition similar to that proposed for papillae in leaves of resistant *mlo* barley plants. Considering that *S. indica* colonization is efficiently arrested in *mlo-*5 mutant plants, we hypothesize that the arsenal of hydrolytic enzymes and effector proteins produced by *S. indica* is not sufficient to overcome altered defenses in *mlo-*5 plants. Additionally, accumulation of toxic compounds at the penetration site in the *mlo-*5 background could decrease the capability of *S. indica* to penetrate the host cell.

The early arrest of *S. indica* colonization in plants carrying the *mlo-*5 allele indicates that the biotrophic phase plays an important role for the establishment of the beneficial barley-endophyte interaction. To test whether a reduction in biotrophic colonization would have an effect on fungal development, we assessed the induction of host root cell death, which is a hallmark of the *S. indica*-plant interaction during later colonization stages, using a well-established vacuolar processing enzyme (VPE) activity assay (Deshmukh et al., 2006; Qiang et al., 2012; Lahrmann et al., 2013). At 10 dpi, root sections of colonized WT and *mlo-*5 plants were collected for total protein extraction. Protein extracts were then incubated with the fluorescent VPE-specific substrate Ac-ESEN-MCA to measure VPE-mediated proteolytic cleavage of MCA. We observed a significant reduction in VPE activity in *S. indica*-treated *mlo-*5 plants compared to WT controls (**Figure 1E**) suggesting a decrease in host cell death. This finding is in agreement with lower colonization levels in *mlo* mutant plants (**Figure 1A**, **Supplementary Figure 1**), Thus, the recessive *mlo* allele effect on the early biotrophic fungal growth also affects the cell death phase.

### Reduced *S. indica* Colonization Correlates With an Early Accumulation of Iron At the Epidermal Layer of *mlo-*5 Mutant Plants

The production of reactive oxygen species (ROS) represents a key factor in pathogen resistance, and has been reported to play a role in a number of beneficial associations (Enkerli et al., 1997; Hückelhoven et al., 1999; Fenster and Hause, 2005; Zuccaro et al., 2011; Lahrmann and Zuccaro, 2012). In addition to their direct antimicrobial effects, ROS have been implicated in the fortification of papillae and are emerging as signaling components during plant immunity (Thordal-Christensen et al., 1997; Torres et al., 2006; Baxter et al., 2014).

**Figure 2:**
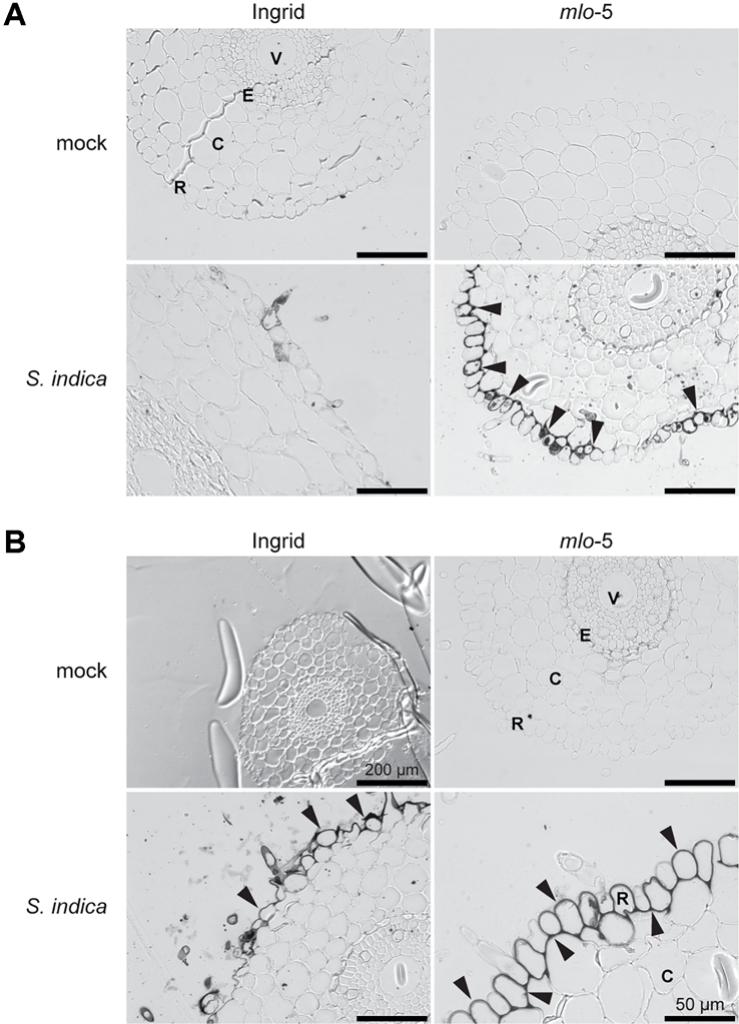
Early accumulation of iron in the epidermal cells of the Ingrid *mlo*-5 mutant upon *S. indica* colonization. Light microscopic pictures show root sections stained with Perls/DAB at 5 **(A)** and 6 dpi **(B)**. Black precipitates, indicative of iron accumulation, appear around root epidermal cells in *mlo*-5 at 5 dpi, whereas iron accumulation in WT plants is only visible after 6 days of *S. indica* colonization (black arrowheads). Size bar = 100 µm unless otherwise indicated. R, rhizodermis; C, cortex; E, endodermis; V, vasculature.

In cereals, the extracellular production of H_2_O_2_ and the resulting oxidative burst in response to biotic stress are dependent on the accumulation of iron (Liu G et al., 2007). To visualize iron accumulation, root tissue of infected and mock-treated plants was stained with Perls/DAB. Consistent with our observations on papillae formation in *mlo-5* mutant plants, iron accumulation was apparent at 5 dpi in *S. indica*-colonized mutant plants (**Figure 2A**), whereas iron depositions started to appear at 6 dpi in WT plants (**Figure 2B**). Iron accumulated at the cell periphery throughout the rhizodermal cell layer, suggesting a systemic reaction of this layer. In leaves it was shown that accumulation of Fe^3+^ occurs specifically around the cell wall appositions just below the site of *Bgh* penetration attempts, where it facilitates H_2_O_2_ production (Greenshields et al., 2007; Liu G et al., 2007), suggesting that the mechanisms underlying iron accumulation and its role in ROS generation may be different in the rhizodermal cell layer. Together, these findings indicate that defense responses are enhanced and occur earlier in the *mlo-*5 mutant resulting in reduced *S. indica* colonization.

### *Mlo* Loss-Of-Function Mutations Result in Enhanced Mycorrhization

**Figure 3:**
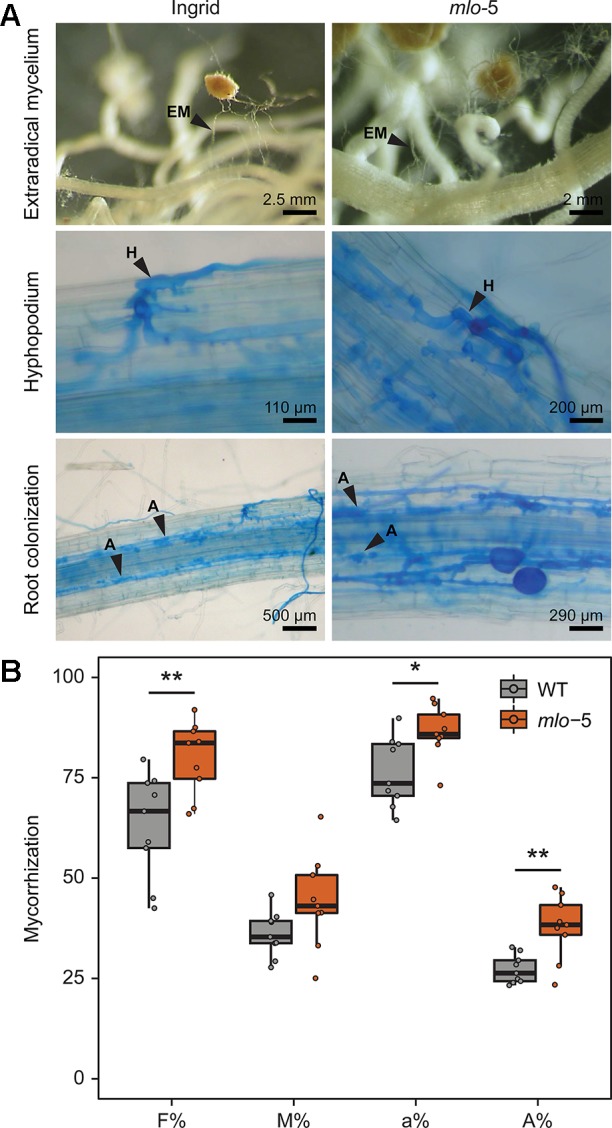
The barley *mlo-*5 allele promotes mycorrhization. Three-day-old seedlings were inoculated with *F. mosseae* inoculum with ≥10 active propagules/g mixed into the soil. After 2 months of cultivation, intraradical colonization was assessed by light microscopy of Cotton blue-stained root tissue in WT and *mlo-*5 plants **(A)**. Black arrowheads indicate extraradical mycelia (EM; upper panel), hyphopodia (H; middle panel), and arbuscules (A; lower panel). **(B)** Mycorrhization was quantified using root tissue stained with fluorescein isothiocyanate-conjugated Wheat Germ Agglutinin (WGA-FITC) following confocal microscopy (*n* = 2). F% reports the percentage of segments showing internal colonization, M% indicates the average percent colonization of root segments, a% quantifies the average presence of arbuscules within the infected areas, A% quantifies the presence of arbuscules in the whole root system. Statistically significant differences in mycorrhization between barley genotypes were determined with Welch two sample *t* tests (*p < 0.5, **p < 0.01).

Like *S. indica*, *F. mosseae* has an intracellular lifestyle. Transcriptome studies comparing mycorrhizal to non-mycorrhizal conditions in *Lotus japonicus* suggested that *MLO* might play a regulatory role in AM colonization (Guether et al., 2009). To test this hypothesis, WT and *mlo-*5 barley plants were inoculated with *F. mosseae* and the rate of AM colonization was determined after 2 months of co-cultivation. Microscopically, we did not observe evident differences in the mycorrhizal phenotype. The extraradical mycelia developed in both plant genotypes (**Figure 3A**, upper panel) and the hyphopodia (**Figure 3A**, middle panel) consisted of similarly swollen hyphae at places where hyphae were in direct contact with the plant epidermal cell. Moreover, arbuscules were produced in cortical cells with a typical branched appearance in colonized WT and *mlo*-5 mutant plants (**Figure 3A**, lower panel). These findings indicate that *MLO* is not essential for the establishment of AM symbiosis and cannot be listed as one of the genes of the AM symbiosis signaling module that are required for the perception of AM-derived signaling molecules (Vigneron et al., 2018). To analyze the first step of the colonization (i.e. the penetration of the epidermal cells) in greater detail, we stained vibratome sections of colonized *mlo*-5 root tissue with WGA-FITC. Confocal microscopy of stained sections revealed that *F. mosseae* follows an intracellular penetration mechanism in the mutant background (**Supplementary Figure 2**), as has been described for *G. margarita* on *Medicago truncatula* roots (Genre et al., 2005; Genre et al., 2008). Similar to *G. margarita*, *F. mosseae* hyphae proliferating from the hyphopodium directly cross the wall of the epidermal cells to which the hyphopodium is adhering (**Supplementary Figure 2**).

We then assessed the success of *F. mosseae* colonization by quantifying the frequency of mycorrhization (F%), the intensity of mycorrhization in the root cortex (M%), the percentage of arbuscules in infected areas (a%), and the presence of arbuscules in the entire root system (A%). In our experiments, F%, a%, and A% were higher in *mlo-*5 mutants compared to WT control plants, while the intensity of mycorrhization did not seem to be affected by the plant phenotype (**Figure 3B**). This is in contrast to the results presented by Ruiz-Lozano and colleagues (1999) who described a negative effect of the *mlo-*5 mutation on *F. mosseae* colonization at 6 weeks. This discrepancy is likely due to differences in inoculum efficiency and the large difference in root material analyzed here (1 m/plant) compared to the earlier study (30 cm/3 plants), or in the time points analyzed. We cannot exclude that colonization of the *mlo-5* mutant plants would be negatively affected at earlier stages, but our data indicate that the absence of this gene is not negatively affecting the symbiosis in the long run. Our observations suggest that *MLO* may be required for optimal AM colonization levels and could be grouped with other so-called downstream genes, which are involved in the re-organization of the plant host cell and facilitate nutrient exchange (Vigneron et al., 2018). While mutations in many of these downstream genes lead to a decrease in colonization success (Wang et al., 2019), the *mlo-*5 allele enhances host susceptibility. *MLO* genes encode plant-specific proteins with seven membrane-spanning domains (Büschges et al., 1997; Devoto et al., 1999; Elliott et al., 2005; Kusch et al., 2016). Despite considerable effort, our knowledge on the biochemical functions of this group of proteins remains limited. However, it has been demonstrated that *mlo*-mediated resistance against *Bgh* relies on actin cytoskeleton function in epidermal cells (Miklis et al., 2007). Similarly, the cytoskeleton is likely involved in the biogenesis of the perifungal membrane, which is an extension of the host plasmalemma surrounding the growing AM intracellular hyphae (Genre and Bonfante, 2002; Genre et al., 2012). Thus, *MLO* may exert control over the number of produced arbuscules through the regulation of cytoskeletal assembly.

## Conclusions

In this study, we show that *MLO* differentially regulates the establishment of beneficial symbioses with the endophyte *S. indica* and the AM fungus *F. mosseae* in barley. During WT colonization by *S. indica* and *F. mosseae*, non-specific defense responses are largely repressed. In contrast, inoculation of *mlo-*5 mutant plants with *S. indica* results in an early activation of strong defense responses, such as accumulation of iron and papillae formation, limiting *S. indica* colonization at the epidermal layer during biotrophic growth and at the onset of the cell death-associated phase, while *F. mosseae* mycorrhization is enhanced in cortex cells at a late colonization stage. Based on these findings, we conclude that the regulatory role of *MLO* may be cell type specific. This hypothesis is in accordance with the recent finding that the *mlo-*5 mutation diminishes *P. palmivora* infection only in young leaf tissue (Le Fevre et al., 2016). Moreover, there seems to be a difference in *mlo* contribution to resistance between monocot and dicot plant species since *mlo* barley and *A. thaliana* mutants display contrasting phenotypes during the interaction with *S. indica*. One explanation could be the prominent role of iron (Fe^3+^)-mediated oxidative (H_2_O_2_) cross-linking of cell wall appositions in barley and other cereals, which does not occur during the *A. thaliana*-*S. indica* interaction (Greenshields et al., 2007; Liu G et al., 2007; Lahrmann et al., 2013). Additionally, the results presented here corroborate the pleiotropic effects of *mlo* in various plant-microbe interactions. Other genes have been shown to display variable regulative roles depending on the colonization strategy of the microbe. For example, in *M. truncatula* the gene *Nod Factor Perception* (*MtNFP*) is essential for the establishment of N-fixing symbiosis, and, as a consequence *Mtnfp* mutants cannot establish the beneficial interaction (Rey et al., 2013). However, *Mtnfp* mutant lines are more susceptible to pathogens including *Aphanomyces euteiches* and *Colletotrichum trifolii*, indicating that the regulation of plant-microbe symbioses is often dependent on the interacting microbe.

## Data Availability Statement

All datasets generated for this study are included in the article/**Supplementary Material**.

## Author Contributions

MH, MN, PB, and AZ conceived and designed experiments. MH, MN, SM, and CG carried out experiments and analyzed the data. MN, HR, PB, and AZ wrote the manuscript.

## Funding

AZ acknowledges support from the Max-Planck-Gesellschaft and the Cluster of Excellence on Plant Sciences (CEPLAS) under Germany’s Excellence Strategy—EXC 2048/1—Project ID: 390686111. Research in Torino was supported by the project 60% (local project of University of Torino) to PB.

## Conflict of Interest

The authors declare that the research was conducted in the absence of any commercial or financial relationships that could be construed as a potential conflict of interest.

The reviewer [RP] declared providing the plant material for this research and confirms the absence of any other collaboration with the authors to the handling editor.
