# Supplementary material for: MLO Differentially Regulates Barley Root Colonization by Beneficial Endophytic and Mycorrhizal Fungi
Source: Front Plant Sci. 2020 Jan 16;10:1678. doi: 10.3389/fpls.2019.01678 (PMC6976535; doi:10.3389/fpls.2019.01678)
Supplement: Supplementary file 1 [file Table_1.docx]

**Supplementary Table 1** Primer sequences used in this study.

| Primer name | Sequence |
| --- | --- |
| *SiTEF*_Fw | TCGTCGCTGTCAACAAGATG |
| *SiTEF*_Rev | ACCGTCTTGGGGTTGTATCC |
| *HvUBI*_Fw | CAGTAGTGGCGGTCGAAGTG |
| *HvUBI*_Rev | ACCCTCGCCGACTACAACAT |
