# Supplementary material for: MLO Differentially Regulates Barley Root Colonization by Beneficial Endophytic and Mycorrhizal Fungi
Source: Front Plant Sci. 2020 Jan 16;10:1678. doi: 10.3389/fpls.2019.01678 (PMC6976535; doi:10.3389/fpls.2019.01678)
Supplement: Supplementary file 2 [file DataSheet_1.pdf]

Supplementary Information for

MLO differentially regulates barley root colonization by beneficial endophytic and mycorrhizal fungi

Magdalena Hilbert[#], Mara Novero[#], Hanna Rovenich[#], Stéphane Mari, Carolin Grimm, Paola Bonfante, Alga Zuccaro*

*Correspondence: Alga Zuccaro, azuccaro@uni-koeln.de
[#]These authors contributed equally.

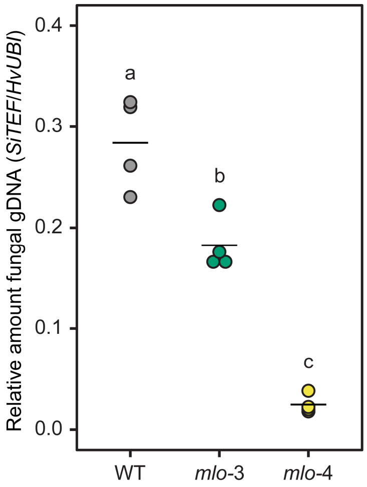

Supplementary Figure 1. Root colonization of different *mlo* mutant lines by *S. indica.* Three-day-old seedlings of wild type, *mlo*-3 and *mlo*-4 barley genotypes were inoculated with a *S. indica* chlamydospore suspension at a final concentration of $5x10^5$ spores/mL in Tween20-water. Tween20-water alone served as mock treatment. Inoculated seedlings were placed into jars containing 1/10 plant nutrition medium. At 10 days post inoculation gDNA was extracted from inoculated root sections as described in the Materials and Methods. Fungal colonization in each biological replicate was confirmed by quantitative PCR (n=4). Letters represent statistically significant differences in colonization according to one-way ANOVA ($F(2,9)=70.47$, $p<0.001$) and Tukey's post-hoc test.

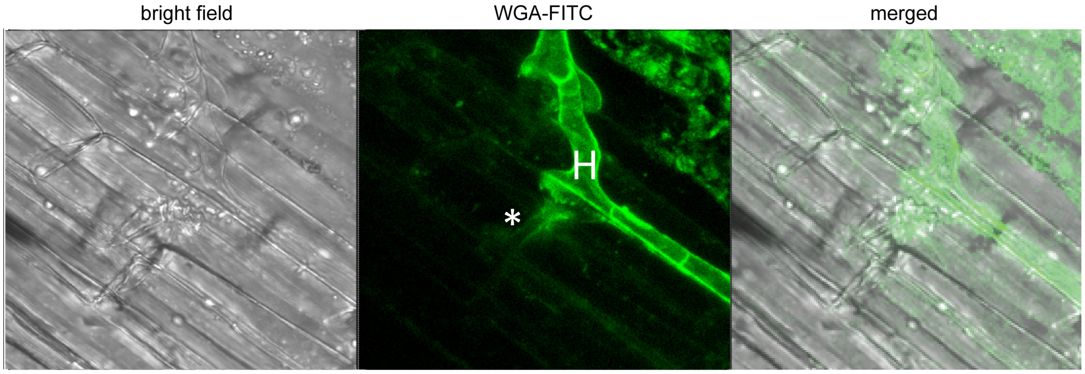

Supplementary Figure 2. ***F. mosseae*** **hyphopodium formation in** ***mlo*-5.** Four 3-day-old seedlings of barley *mlo*-5 were treated with *F. mosseae* inoculum. After two months of cultivation, one half of the root apparatus was treated with Wheat Germ Agglutinin (WGA) conjugated with the fluorescent probe fluorescein isothiocyanate (FITC) to analyze fungal penetration in the root epidermal cells by confocal microscopy. H: hyphopodium. * intracellular hyphae.
